# Major depressive disorder in multiple sclerosis associated with differences in disease modifying therapy and demographics

**DOI:** 10.3389/fneur.2025.1663778

**Published:** 2025-09-15

**Authors:** Nathanael J. Lee, Gavin Hui, C. William Pike, Kristin Galetta, Lucas B. Kipp, Jeffrey Dunn, Scheherazade Le, S. Sai Folmsbee

**Affiliations:** ^1^Division of Neuroimmunology and Neurological Infections, Department of Neurology, School of Medicine, Johns Hopkins University, Baltimore, MD, United States; ^2^Department of Neurology and Neurological Sciences, Stanford University, Stanford, CA, United States; ^3^Atropos Health, New York, NY, United States; ^4^Department of Psychiatry and Behavioral Sciences, Stanford University, Stanford, CA, United States

**Keywords:** multiple sclerosis, demographics, disease modifying therapeutics, depression, epidemiology

## Abstract

Persons with multiple sclerosis (pwMS) are often diagnosed with major depressive disorder (MDD). However, there is a paucity of knowledge regarding the association between different demographic features and such co-diagnosis, as well as the clinical implications the co-diagnosis may carry. This study investigated whether specific demographics demonstrated any correlation with co-diagnosis of MS and MDD, and how MDD comorbidity may potentially impact clinical outcomes. In this single-center study, Black pwMS were more likely to have a MDD comorbidity, and Hispanic pwMS were less likely. MDD comorbidity in pwMS was associated with significantly increased time to disease-modifying therapy (DMT), with the greatest increase in time associated with individuals who received the MDD diagnosis after the MS diagnosis. Among inpatient pwMS, individuals with MDD comorbidity were associated with a decreased usage of MRI while hospitalized. Those who received MDD diagnosis prior to MS were associated with an even further decreased usage of inpatient MRI, and greater mortality. These findings suggest that patient demographics play an important role in how clinicians diagnose MDD in patients with MS. Furthermore, co-diagnosis of MDD may be an important variable that affects healthcare resource utilization and health outcomes.

## Introduction

Multiple sclerosis (MS) is an inflammatory demyelinating disease of the central nervous system (CNS) affecting millions of patients worldwide (1). As the global prevalence, incidence, and disease burden have been increasing in recent years (2), a higher number of associated comorbidities with MS are being elucidated. These include psychiatric disorders such as major depressive disorder (MDD) (3, 4), which were specifically identified as the most common comorbidity in persons with MS (pwMS) (5). Nearly 50% of pwMS report having MDD, which is at least 3 to 10 times greater than that of the general population (6, 7). MDD symptoms are also strongly associated with increase in MS symptoms, such as fatigue (8), as well as overall health-related quality of life (9) and greater disability (10).

Furthermore, for pwMS, having at least one psychiatric disorder, including MDD, has been associated with an increased hazard of evidence of disease activity (5), highlighting the importance of MDD as a comorbidity in this patient population. Symptoms associated with MDD have been associated with one of the first symptoms described in pwMS (11), and has also been identified as one of the prodromal symptoms in MS disease course (12). Finally, there is growing evidence that MDD in MS may represent a unique syndrome, separate from standard MDD, with a novel pathogenesis and symptomology (13).

Despite such high prevalence of MS and MDD co-diagnosis, the exact underlying pathobiological mechanism is unknown. Such investigation also becomes exponentially difficult given that MS is a highly heterogeneous disease, with various disease courses and outcomes (14–18). Recent studies have shown that social demographic features, including socioeconomic, non-medical factors influencing health outcomes, are strongly associated with disability accrual in pwMS, potentially contributing to the heterogeneity of MS (19–21). For example, previous studies have identified worse clinical outcomes in Black, Hispanic, and Latinx pwMS compared to White pwMS (22, 23). Furthermore, some studies have shown that there is a discrepancy in clinical outcomes among different sexes, mainly that female pwMS have worse disease courses (23), although some studies suggest the opposite, or mixed outcomes (24).

Importantly, the prevalence of MDD has also been associated with sociodemographic features (25, 26). Although there exists an intricate association between MDD and MS, as well as implications of such variables in disease outcomes in both MS and MDD, there is a paucity of knowledge regarding how they impact the prevalence of MS and MDD co-diagnosis and its potential clinical implications, such as delayed diagnosis of either condition. Delayed diagnosis of MS can also lead to the heterogeneity of the disease course, as delayed time to initiating disease-modifying therapies (DMTs) is associated with poorer clinical outcomes in pwMS (19–21). The overarching goal of the study was to investigate how MDD comorbidity and specific demographics demonstrated any association with MS treatment and outcomes.

## Methods

### Data collection

This was a single-center study performed at Stanford University Hospital. We evaluated patients with electronic medical records between 2008 and 2024 with the diagnosis of MS and/or MDD using the International Classification for Disease versions 9 and 10 (ICD9, ICD10), ages above 18. Detailed methodology has been previously published (27). Patients’ demographic data, including race (White, Black, Asian, or Other/Unknown) and self-identification as Hispanic, age, and biological sex (male or female) were identified. For the inpatient data, inclusion criteria were the first inpatient or emergency department visit after initial MS diagnosis. Within the co-diagnosis cohort, we also subdivided the group into which pwMS had the diagnosis of MDD before or after the diagnosis of MS. Within this population of pwMS, primary outcomes were as follows: usage of magnetic resonance imaging (MRI) of any part of the neuraxis, time to MRI from admission time, hospital length of stay, discharge to outpatient follow-up time frame, prevalence of pwMS on DMTs, and time-to-DMT-use after MS diagnosis. Charlson Comorbidity index score and mortality data (although specific causes of death were not available) were also calculated for each patient and reported as cohort summary statistics.

### Statistical analyses

A two-sided *p*-value of 0.05 was set as the threshold for statistical significance. All analyses were performed using R version 4.2 on the Atropos Health platform (27). All data collection was approved by the Stanford University Institutional Review Board.

## Results

### Demographics overview of co-diagnosis of MS and MDD

A total of 4,554 pwMS only, and 292 pwMS and MDD, were identified, demonstrating that 6.0% of pwMS were also diagnosed with MDD. Of those, 35.3% (*n* = 97) had the diagnosis of MDD prior to MS, and 67.7% (*n* = 178) afterwards. For inpatient individuals, a total of 1,031 pwMS only, and 134 pwMS and MDD were identified, demonstrating that 11.5% of inpatient pwMS were also diagnosed with MDD. Of those, 27.6% (*n* = 35) had the diagnosis of MDD prior to MS, and 72.4% (*n* = 92) afterwards.

### Sex and co-diagnosis of MS and MDD

There was no significant difference in the proportion of pwMS with or without the co-diagnosis of MDD regarding sex [74.2% of female pwMS only (*n* = 3,381) vs. 81.2% of female pwMS with MDD (*n* = 2,377)]. However, among inpatient individuals, female pwMS had a trend toward a decreased association with a diagnosis of MDD after MS diagnosis (83.7% before vs. 71.4% after), and male pwMS had an increased association with a diagnosis of MDD before MS diagnosis (28.6% vs. 16.3%), although this difference was not statistically significant (*p* = 0.14).

### DMT treatment and race and ethnicity

**Figure 1:**
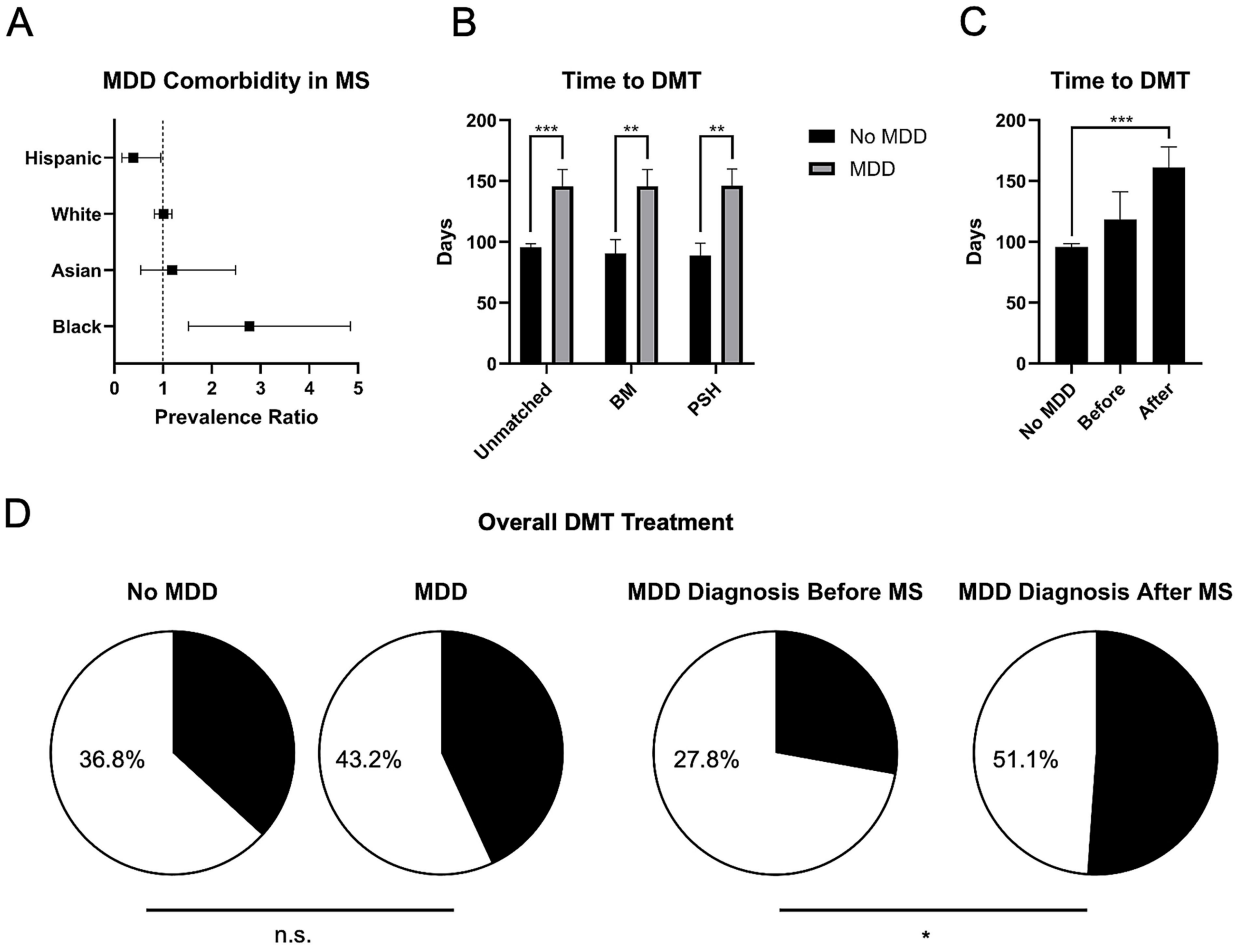
Differences in DMT usage among pwMS and MDD comorbidity. **(A)** Among those who received DMT within 1 year of MS diagnosis, Black pwMS showed increased association with MDD comorbidity by prevalence ratio (PR), and Hispanic pwMS showed a decreased associated with MDD comorbidity by PR. **(B)** pwMS with MDD were associated with a significantly increased time to DMT usage, even after correcting for sex and age by basic matching (BM), and propensity score matching (PSM). **(C)** When subdivided into those pwMS who received the co-diagnosis of MDD before and after MS diagnosis, the greatest increase in time to DMT treatment was found in those who received the co-diagnosis after. **(D)** Although there was no significant difference in the proportion of pwMS getting overall DMT treatment when compared to those with MDD comorbidity (left), pwMS who received the MDD diagnosis afterwards were associated with an overall increased DMT usage when compared to those who received the diagnosis before (right). ^*^p < 0.05, ^**^p < 0.01, and ^***^p < 0.001, n.s., not significant.

A total of 1,047 pwMS only, and 67 pwMS and MDD, were identified who were treated with DMTs within 1 year of diagnosis, demonstrating that 6.0% of pwMS treated with DMTs were also diagnosed with MDD. Among this group, there was significant difference in the proportion of pwMS with or without the co-diagnosis MDD with regards to race and ethnicity. Specifically, 15.0% (*n* = 11/73) of Black pwMS carried the co-diagnosis, whereas only 6.1% (*n* = 43/709) of White pwMS, 7.1% (*n* = 6/85) of Asian pwMS, and 2.8% (*n* = 7/247) of pwMS with Other race had the co-diagnosis. Additionally, pwMS identifying as Hispanic only had 2.4% (*n* = 4/164) with an MDD co-diagnosis. When analyzing these by prevalence ratio (PR), Black pwMS were found to be more likely to be associated with MDD comorbidity (PR 2.77, 95% CI 1.52–4.85), and Hispanic pwMS were found to be less likely to be associated with MDD comorbidity (PR 0.39, 95% CI 0.15–0.95) (Figure 1A). There was no significant difference found for white pwMS (PR 1.01, 95% CI 0.82–1.18) or Asian pwMS (PR 1.19, 95% CI 0.54–2.49) (Figure 1A).

### DMT and co-diagnosis of MS and MDD

There was no significant difference in the proportion of pwMS receiving DMTs in this group, when comparing those without MDD co-diagnosis and those with MDD co-diagnosis (23.67% vs. 23.63%, respectively). However, regarding the time to receive DMTs, pwMS and MDD had a longer time to prescription (95.68 vs. 145.83 days, for those without and with MDD comorbidity, respectively, *p* < 0.001) (Figure 1B). This difference persisted even after basic matching (BM, *p* < 0.01), correcting for age and sex, and for propensity score matching (PSM, *p* < 0.01) (Figure 1B). Furthermore, among pwMS and MDD, individuals diagnosed with MDD after MS had longer times to receiving DMTs (161.22 days, *p* < 0.001) (Figure 1C). When comparing overall DMT usage, there was no significant difference between the proportion of pwMS when comparing those without and those with MDD comorbidity (36.8% vs. 43.2%, respectively) (Figure 1D, left). However, among pwMS and MDD, individuals diagnosed with MDD after MS had a significantly increased proportion of receiving DMTs overall (27.84% vs. 51.12%, for MDD diagnosed before and after, respectively, *p* < 0.05) (Figure 1D, right).

### Co-diagnosis of MS and MDD and inpatient outcomes

**Figure 2:**
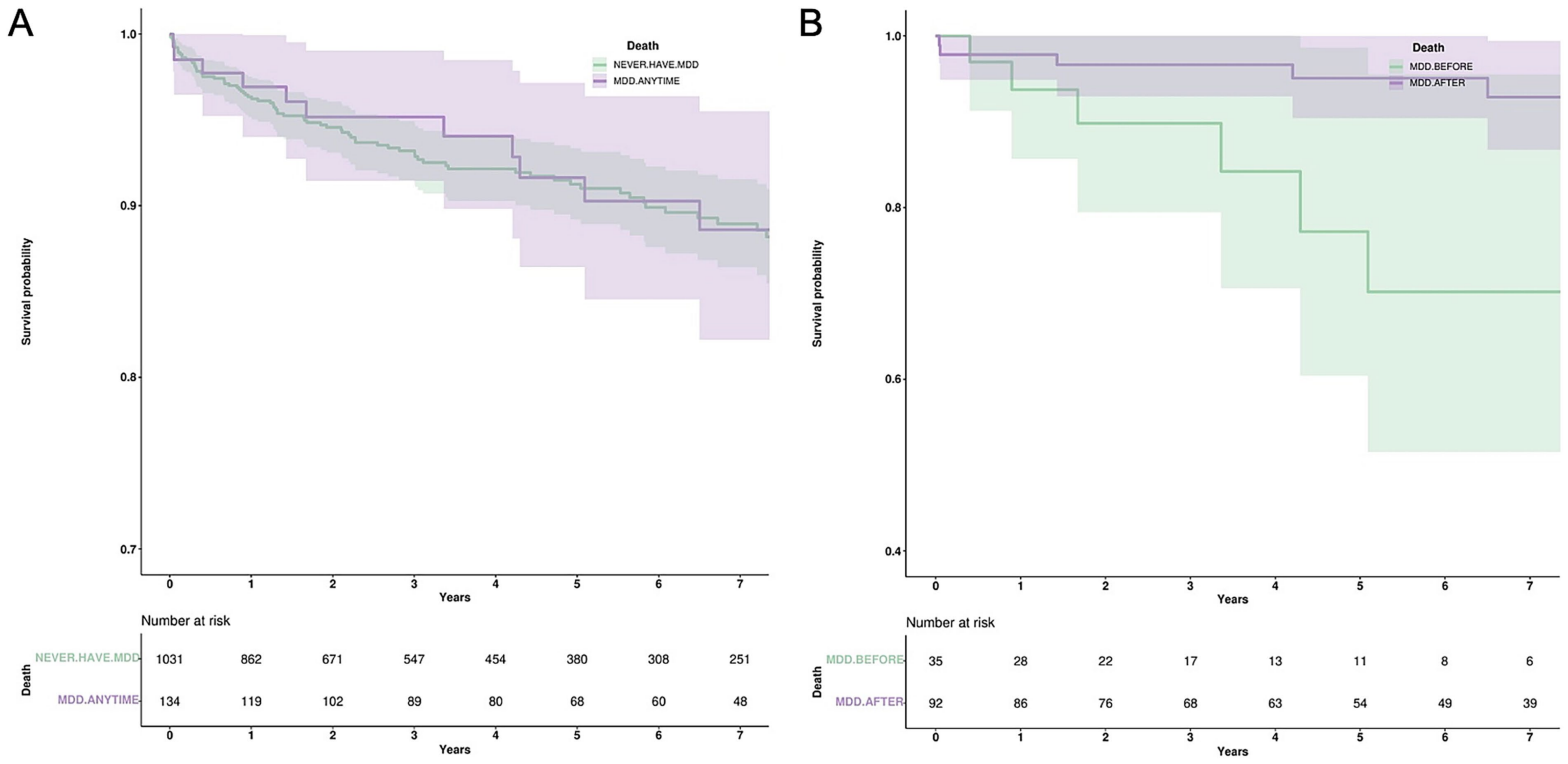
Differences in mortality among pwMS and MDD comorbidity is affected by the timing of diagnosis. Kaplan–Meier survival curves are shown above to compare the impact of MDD comorbidity on pwMS. **(A)** Among hospitalized pwMS, there was no significant difference in mortality when comparing patients with MDD comorbidity to those without (*p* = 0.64). **(B)** However, when comparing the timing of MDD comorbidity, those diagnosed with MDD prior to MS showed were associated with an increased mortality when compared to those who were diagnosed after (*p* < 0.05).

Overall, pwMS and MDD had lower utilization of MRI during admission (18.43% vs. 8.96%, without and with MDD, respectively, *p* < 0.01). Specifically, pwMS and MDD prior to the diagnosis of MS had a lower utilization of inpatient MRI compared to pwMS with MDD diagnosis after MS diagnosis (2.86% vs. 11.96%, *p* = 0.18), although it was not statistically significant. Similarly, pwMS with the co-diagnosis of MDD had a trend towards a faster time to MRI from admission, (17.3 vs. 7.44 h, for those without and with MDD, respectively, *p* = 0.11). Finally, there were no significant differences found for mean hospital length of stay (4.82 vs. 3.78 days), time to first outpatient visit (135.5 vs. 118.5 days), or death (8.15% vs. 8.21%) (Figure 2A) when comparing those without and with MDD comorbidity. Similarly, when comparing those who received the MDD diagnosis before MS with those who received it after, there was no significant difference in mean hospital length of stay (2.46 vs. 3.71 days) or time to first outpatient visit (87.8 vs. 124.5 days). However, those who received the MDD diagnosis before MS had a significantly increased risk of death when compared to those who received the diagnosis after (17.15% vs. 5.43%, *p* = 0.015) (Figure 2B).

Furthermore, inpatient pwMS with MDD had overall worse comorbidity score (4.3 vs. 4.0, without and with MDD, respectively), notably including the following comorbidity prevalence differences: diabetes (12.12% vs. 21.64%); chronic pulmonary disease (18.82% vs. 35.82%); and mild liver disease (9.89% vs. 18.66%). Additionally, pwMS diagnosed with MDD before MS had a higher comorbidity score when compared to those diagnosed after (5.9 vs. 3.4).

## Discussion

MDD is a common comorbidity in pwMS, affecting greater than 6% of the overall population, and over 11% of hospitalized pwMS, based on our single-center study, and up to 50% in previous studies (6, 7). We demonstrated a strong association between specific SDoH and the diagnosis and treatment of MS and MDD. Overall, Black pwMS had the highest prevalence of co-diagnosis, with a prevalence ratio of nearly three times that of the patient population. Conversely, Hispanic pwMS had the lowest prevalence, with a significantly decreased prevalence ratio of less than half of the overall population. Furthermore, although there was no significant difference between male and female pwMS, there was a trend towards more male patients having the diagnosis of MDD before that of MS when compared to female patients.

The above findings show that there are strong associations among patient demographics, especially race/ethnicity and both the co-diagnosis of MS and MDD, as well as the timing of MDD diagnosis in relation to that of MS. Specifically, we demonstrated that Black pwMS carry this co-diagnosis more often. This observation is particularly concerning, given such co-diagnosis was associated with worse health outcomes in both inpatient and outpatient settings. These data are consistent with previous data suggesting increased stress indicators in Black pwMS (28) and raise new concerns for potential treatment disparities, given that a previous study demonstrated that Black pwMS may also be less likely to receive antidepressant treatment (27).

Regarding Hispanic pwMS, previous studies have suggested that they have differences in disability associated with MS (29). Thus, it is surprising that Hispanic pwMS had MDD comorbidity less frequently. However, this lack of MDD diagnosis may not reflect decreased depressive symptoms, but rather may be due to lack of access to psychiatric resources, as previous studies have shown Hispanic pwMS with insufficient access to mental health services (30). Furthermore, there may be other cultural aspects, including stigma towards depression and seeking help, as previously described in Hispanic individuals (31–33). Additionally, there may be other factors, just not race/ethnicity, that may play a role in this finding, including socioeconomic stability and healthcare accessibility.

For pwMS admitted to the hospital, we also demonstrated that co-diagnosis of MDD was associated with significantly different inpatient resource utilization. Although pwMS with MDD overall had lower utilization of MRI during admission, especially if they had MDD diagnosis prior to that of MS, there was a surprising trend toward receiving the scans more quickly. PwMS with MDD also had a longer time to initial DMT prescription, especially those who had MDD diagnosis after MS, who also had a higher proportion of receiving DMTs.

The causes of the difference in MRI utilization between groups remains unclear, although multiple clinical factors may be indicated. For example, individuals with MDD may be more likely to present to the hospital for non-MS related symptoms, and thus they may have fewer indications for imaging. Conversely, given the vulnerability of this population and the other findings of this study regarding delays in DMT, there may also be concern that individuals with MDD may be receiving inadequate imaging while inpatient. Whatever the cause, these data demonstrate the importance of continuing to investigate differences in clinical management in pwMS with MDD.

The timing of MDD diagnosis in relation to MS diagnosis demonstrates complicated results, further providing evidence that MDD in MS may represent a unique entity when compared to traditional MDD. Regarding overall mortality, earlier MDD diagnosis portended a poorer outcome. However, later MDD diagnosis was also associated with other poorer outcomes, including worsening delay to initiation of DMT. Together, these suggest that this area requires further investigation, especially given the fact that both MS and MDD are chronic diseases, and that the current study does not have access to specific causes of death in our cohort. It is possible that MDD may be an early symptom of MS itself, or that having a debilitating, chronic disorder such as MS may increase the risk of development of the disorder (34). Alternatively, it may also represent a different pathobiological mechanism of MS, as MS is a highly heterogeneous disease.

This study has some important limitations. First, this study does not include the exact temporality of the diagnoses and comorbidity; for example, knowing how many days or months the diagnoses are separated by may clarify their relationship. Additionally, this study only included those with formal, recorded MDD diagnoses, which may explain the relatively low prevalence detected in this sample when compared to previous studies of depressive symptoms in MS. The current study also does not have information regarding what treatments each pwMS may be receiving for their MDD. Another limitation of the study is that both MS and MDD are highly heterogeneous disorders, with a wide range of symptomatology and associated disability. Therefore, it is difficult to generalize the association between the two diagnoses. Furthermore, for mortality data, we have not investigated the specific causes of death, which limits our interpretation. Lastly, interpretating demographics data is inherently challenging, especially given the complex, intricate relationship it may have with other features affecting healthcare outcomes, including healthcare accessibility, socioeconomic barriers, and other variables, which can limit the conclusions from these data. Future study may improve on this work by incorporating patient-centered outcomes to better delineate the complex interaction MS and MDD may have.

Despite the limitations, our data suggest that MDD and MS have an intricate association, especially in the context of patient demographics. Our study underlines crucial role they play in both patient experiences, as well as inpatient and outpatient outcomes.

## Data Availability

The raw data supporting the conclusions of this article will be made available by the authors, without undue reservation.

## References

[1] Reich DS Lucchinetti CF Calabresi PA. Multiple sclerosis. N Engl J Med. (2018) 378:169–80. doi: 10.1056/NEJMra1401483, PMID: 29320652 PMC6942519

[2] Qian Z Li Y Guan Z Guo P Zheng K Du Y. Global, regional, and national burden of multiple sclerosis from 1990 to 2019: findings of global burden of disease study 2019. Front Public Health. (2023) 11:1073278. doi: 10.3389/fpubh.2023.1073278, PMID: 36875359 PMC9982151

[3] Machado EFA Glehn FV Sasaki J Tauil CB David ACD. Depression and sedentary behaviour in women with multiple sclerosis. Mult Scler Relat Disord. (2024) 91:105895. doi: 10.1016/j.msard.2024.105895, PMID: 39342813

[4] Boeschoten RE Braamse AMJ Beekman ATF Cuijpers P van Oppen P Dekker J. Prevalence of depression and anxiety in multiple sclerosis: a systematic review and meta-analysis. J Neurol Sci. (2017) 372:331–41. doi: 10.1016/j.jns.2016.11.067, PMID: 28017241

[5] Salter A Lancia S Kowalec K Fitzgerald KC Marrie RA. Comorbidity and disease activity in multiple sclerosis. JAMA Neurol. (2024) 81:1170–7. doi: 10.1001/jamaneurol.2024.2920, PMID: 39291661 PMC11411448

[6] Caine ED Schwid SR. Multiple sclerosis, depression, and the risk of suicide. Neurology. (2002) 59:662–3. doi: 10.1212/WNL.59.5.662, PMID: 12221154

[7] Feinstein A. Multiple sclerosis and depression. Mult Scler. (2011) 17:1276–81. doi: 10.1177/1352458511417835, PMID: 22058085

[8] Knowles LM Mistretta EG Arewasikporn A Hugos CL Cameron MH Haselkorn JK. Improvement in depressive symptoms is associated with sustained improvement in fatigue impact in adults with multiple sclerosis. Mult Scler Relat Disord. (2024) 92:106158. doi: 10.1016/j.msard.2024.106158, PMID: 39577297 PMC11737376

[9] Iaquinto S Ineichen BV Salmen A Kuhle J Benkert P Hofer L. Factors associated with low health-related quality of life in persons with multiple sclerosis: a quantile-based segmentation approach. PLoS One. (2024) 19:e0312486. doi: 10.1371/journal.pone.0312486, PMID: 39570987 PMC11581332

[10] Frank HA Chao M Tremlett H Marrie RA Lix LM McKay KA. Comorbidities and their association with outcomes in the multiple sclerosis population: a rapid review. Mult Scler Relat Disord. (2024) 92:105943. doi: 10.1016/j.msard.2024.105943, PMID: 39489083

[11] Butler MA Bennett TL. In search of a conceptualization of multiple sclerosis: a historical perspective. Neuropsychol Rev. (2003) 13:93–112. doi: 10.1023/A:1023884322540, PMID: 12887041

[12] Chertcoff AS Yusuf FLA Zhu F Evans C Fisk JD Zhao Y. Psychiatric comorbidity during the prodromal period in patients with multiple sclerosis. Neurology. (2023) 101:e2026–34. doi: 10.1212/WNL.0000000000207843, PMID: 37748884 PMC10662981

[13] Hasselmann H Bellmann-Strobl J Ricken R Oberwahrenbrock T Rose M Otte C. Characterizing the phenotype of multiple sclerosis-associated depression in comparison with idiopathic major depression. Mult Scler. (2016) 22:1476–84. doi: 10.1177/1352458515622826, PMID: 26746809

[14] Sormani MP Chataway J Kent DM Marrie RA. Assessing heterogeneity of treatment effect in multiple sclerosis trials. Mult Scler. (2023) 29:1158–61. doi: 10.1177/13524585231189673, PMID: 37555493 PMC10413777

[15] Lucchinetti C Brück W Parisi J Scheithauer B Rodriguez M Lassmann H. Heterogeneity of multiple sclerosis lesions: implications for the pathogenesis of demyelination. Ann Neurol. (2000) 47:707–17. doi: 10.1002/1531-8249(200006)47:6<707::AID-ANA3>3.0.CO;2-Q, PMID: 10852536

[16] Klineova S Lublin FD. Clinical course of multiple sclerosis. Cold Spring Harb Perspect Med. (2018) 8:a028928. doi: 10.1101/cshperspect.a028928, PMID: 29358317 PMC6120692

[17] Lublin FD Häring DA Ganjgahi H Ocampo A Hatami F Čuklina J. How patients with multiple sclerosis acquire disability. Brain. (2022) 145:3147–61. doi: 10.1093/brain/awac016, PMID: 35104840 PMC9536294

[18] Filippi M Bar-Or A Piehl F Preziosa P Solari A Vukusic S. Multiple sclerosis. Nat Rev Dis Primers. (2018) 4:43. doi: 10.1038/s41572-018-0041-4 30410033

[19] Giovannoni G Butzkueven H Dhib-Jalbut S Hobart J Kobelt G Pepper G. Brain health: time matters in multiple sclerosis. Mult Scler Relat Disord. (2016) 9:S5–S48. doi: 10.1016/j.msard.2016.07.003, PMID: 27640924

[20] Filippi M Amato MP Centonze D Gallo P Gasperini C Inglese M. Early use of high-efficacy disease-modifying therapies makes the difference in people with multiple sclerosis: an expert opinion. J Neurol. (2022) 269:5382–94. doi: 10.1007/s00415-022-11193-w, PMID: 35608658 PMC9489547

[21] Noyes K Weinstock-Guttman B. Impact of diagnosis and early treatment on the course of multiple sclerosis. Am J Manag Care. (2013) 19:s321–31. PMID: 24494633

[22] Jacobs BM Peter M Giovannoni G Noyce AJ Morris HR Dobson R. Towards a global view of multiple sclerosis genetics. Nat Rev Neurol. (2022) 18:613–23. doi: 10.1038/s41582-022-00704-y, PMID: 36075979

[23] Moody SN Manuel M Willette A Shirtcliff E Copeland B Lovera J. The intersection of race and sex on the clinical and cognitive progression of multiple sclerosis. J Neurol Sci. (2024) 466:123260. doi: 10.1016/j.jns.2024.123260, PMID: 39476715 PMC11587816

[24] Kister I Bacon T Cutter GR. How multiple sclerosis symptoms vary by age, sex, and race/ethnicity. Neurol Clin Pract. (2021) 11:335–41. doi: 10.1212/CPJ.0000000000001105, PMID: 34476125 PMC8382423

[25] Wilkinson LL Long-Daniels A Appah M Zhai Y Watson DM Walker K. The association between social determinants of health and depressive disorders: a 2017 behavioral risk factor surveillance system (BRFSS) analysis. Psychiatry Int. (2023) 4:147–59. doi: 10.3390/psychiatryint4020017

[26] Kirkbride JB Anglin DM Colman I Dykxhoorn J Jones PB Patalay P. The social determinants of mental health and disorder: evidence, prevention and recommendations. World Psychiatry. (2024) 23:58–90. doi: 10.1002/wps.21160, PMID: 38214615 PMC10786006

[27] Sai Folmsbee S Hui G Yuan Y Gombar S Han M Le S. Antipsychotic medications associated with increased length of hospital stay in autoimmune encephalitis and multiple sclerosis: a retrospective study. J Clin Neurosci. (2024) 124:87–93. doi: 10.1016/j.jocn.2024.04.021, PMID: 38677201

[28] Hunter EA Meyer JM Brown GM Hanks MA. Stress indicators in minorities with multiple sclerosis. Mult Scler Relat Disord. (2023) 78:104914. doi: 10.1016/j.msard.2023.104914, PMID: 37499341

[29] Silveira SL Motl RW Marquez DX Lancia S Salter A. Physical activity as a correlate of symptoms, quality of life, comorbidity, and disability status in Hispanics with multiple sclerosis – PubMed. Disabil Health J. (2022) 16:101398. doi: 10.1016/j.dhjo.2022.101398 36402726

[30] Buchanan RJ Zuniga MA Carrillo-Zuniga G Chakravorty BJ Tyry T Moreau RL. A pilot study of Latinos with multiple sclerosis: demographic, disease, mental health, and psychosocial characteristics. J Soc Work Disabil Rehabil. (2011) 10:211–31. doi: 10.1080/1536710X.2011.622959, PMID: 22126140

[31] Turan JM Elafros MA Logie CH Banik S Turan B Crockett KB. Challenges and opportunities in examining and addressing intersectional stigma and health. BMC Med. (2019) 17:7. doi: 10.1186/s12916-018-1246-9, PMID: 30764816 PMC6376691

[32] Mascayano F Tapia T Schilling S Alvarado R Tapia E Lips W. Stigma toward mental illness in Latin America and the Caribbean: a systematic review. Braz J Psychiatry. (2016) 38:73–85. doi: 10.1590/1516-4446-2015-1652, PMID: 27111703 PMC7115468

[33] Yang LH Thornicroft G Alvarado R Vega E Link BG. Recent advances in cross-cultural measurement in psychiatric epidemiology: utilizing ‘what matters most’ to identify culture-specific aspects of stigma. Int J Epidemiol. (2014) 43:494–510. doi: 10.1093/ije/dyu039, PMID: 24639447 PMC13387039

[34] Lotfaliany M Bowe SJ Kowal P Orellana L Berk M Mohebbi M. Depression and chronic diseases: co-occurrence and communality of risk factors. J Affect Disord. (2018) 241:461–8. doi: 10.1016/j.jad.2018.08.011, PMID: 30149333

